# Supplementary material for: Mining Google and Apple mobility data: temporal anatomy for COVID-19 social distancing
Source: Sci Rep. 2021 Feb 18;11:4150. doi: 10.1038/s41598-021-83441-4 (PMC7892828; doi:10.1038/s41598-021-83441-4)
Supplement: Supplementary file 1 — Supplementary Information. [file 41598_2021_83441_MOESM1_ESM.pdf]

Mining Google and Apple mobility data: Temporal Anatomy for COVID-19 Social Distancing

Corentin Cot[1,2,+], Giacomo Cacciapaglia[1,2,*,+], and Francesco Sannino[3,4,+]

[1]Institut de Physique des 2 Infinis (IP2I), CNRS/IN2P3, UMR5822, 69622 Villeurbanne, France
[2]Université de Lyon, Université Claude Bernard Lyon 1, 69001 Lyon, France
[3]CP3-Origins & the Danish Institute for Advanced Study, University of Southern Denmark, Campusvej 55, DK-5230 Odense, Denmark
[4]Dipartimento di Fisica E. Pancini, Università di Napoli Federico II & INFN sezione di Napoli, Complesso Universitario di Monte S. Angelo Edificio 6, via Cintia, 80126 Napoli, Italy
[*]g.cacciapaglia@ipnl.in2p3.fr
[+]these authors contributed equally to this work

ABSTRACT

We employ the Google and Apple mobility data to identify, quantify and classify different degrees of social distancing and characterise their imprint on the first wave of the COVID-19 pandemic in Europe and in the United States. We identify the period of enacted social distancing via Google and Apple data, independently from the political decisions. Our analysis allows us to classify different shades of social distancing measures for the first wave of the pandemic. We observe a strong decrease in the infection rate occurring two to five weeks after the onset of mobility reduction. A universal time scale emerges, after which social distancing shows its impact. We further provide an actual measure of the impact of social distancing for each region, showing that the effect amounts to a reduction by $20\% - 40\%$ in the infection rate in Europe and $30\% - 70\%$ in the US.

Supplementary figures and tables

Fit parameters: first wave and 6-week average mobility variations in Europe								
Country	ISO	a	γ	b	Work.	Res.	Driv.	Walk.
France	FRA	7.641(5)	0.690(8)	$1.20(3) \times 10^3$	−65	28	−73	−82
Italy	ITA	8.185(11)	0.536(12)	106(3)	−62	29	−71	−76
Spain	ESP	8.432(6)	0.769(11)	$1.88(5) \times 10^3$	−66	28	−80	−87
United Kingdom	GBR	8.293(8)	0.407(6)	65(2)	−59	23	−58	−55
Germany	DEU	7.580(7)	0.722(12)	$1.58(4) \times 10^3$	−39	14	−43	−40
Denmark	DNK	7.591(13)	0.458(11)	42(2)	−45	15	−29	−35
Switzerland	CHE	8.142(3)	0.794(8)	$2.45(4) \times 10^3$	−45	20	−40	−40
Sweden	SWE	8.07(3)	0.392(9)	52.6(7)	−28	10	−9.1	−27
Slovakia	SVK	6.00(6)	0.44(2)	139(7)	−45	17	−46	−47
Portugal	PRT	7.90(1)	0.648(14)	$9.5(4) \times 10^2$	−59	30	−75	−84
Poland	POL	6.01(2)	0.588(15)	$9.6(4) \times 10^2$	−41	17	−59	−70
Ireland	IRL	8.541(7)	0.612(11)	$9.8(5) \times 10^2$	−60	25	−61	−66
Netherlands	NLD	7.884(6)	0.555(7)	277(5)	−43	16	−48	−47
Romania	ROU	6.877(17)	0.461(12)	176(8)	−46	17	−66	−73
Bulgaria	BGR	6.13(2)	0.326(8)	40(2)	−42	16	−64	−68
Croatia	HRV	6.25(1)	0.78(2)	$4.7(2) \times 10^3$	−52	19	−66	−64
Norway	NOR	7.261(6)	0.710(13)	$0.84(2) \times 10^3$	−46	16	−30	−37
Austria	AUT	7.410(6)	1.07(2)	$5.9(2) \times 10^4$	−53	20	−56	−64
Hungary	HUN	5.961(11)	0.55(1)	$6.5(3) \times 10^2$	−42	17	−48	−69
Serbia	SRB	7.399(7)	0.658(9)	$2.68(9) \times 10^3$	−59	20	−68	−71
Belgium	BEL	8.498(8)	0.585(8)	338(7)	−57	24	−56	−43

Table T1. First wave fits and average mobility reductions in Europe. Values of the fit for a, b and γ for the first wave in the 21 European countries considered in this study, together with the 95% CL error. Six week average mobility reduction for Google and Apple categories.

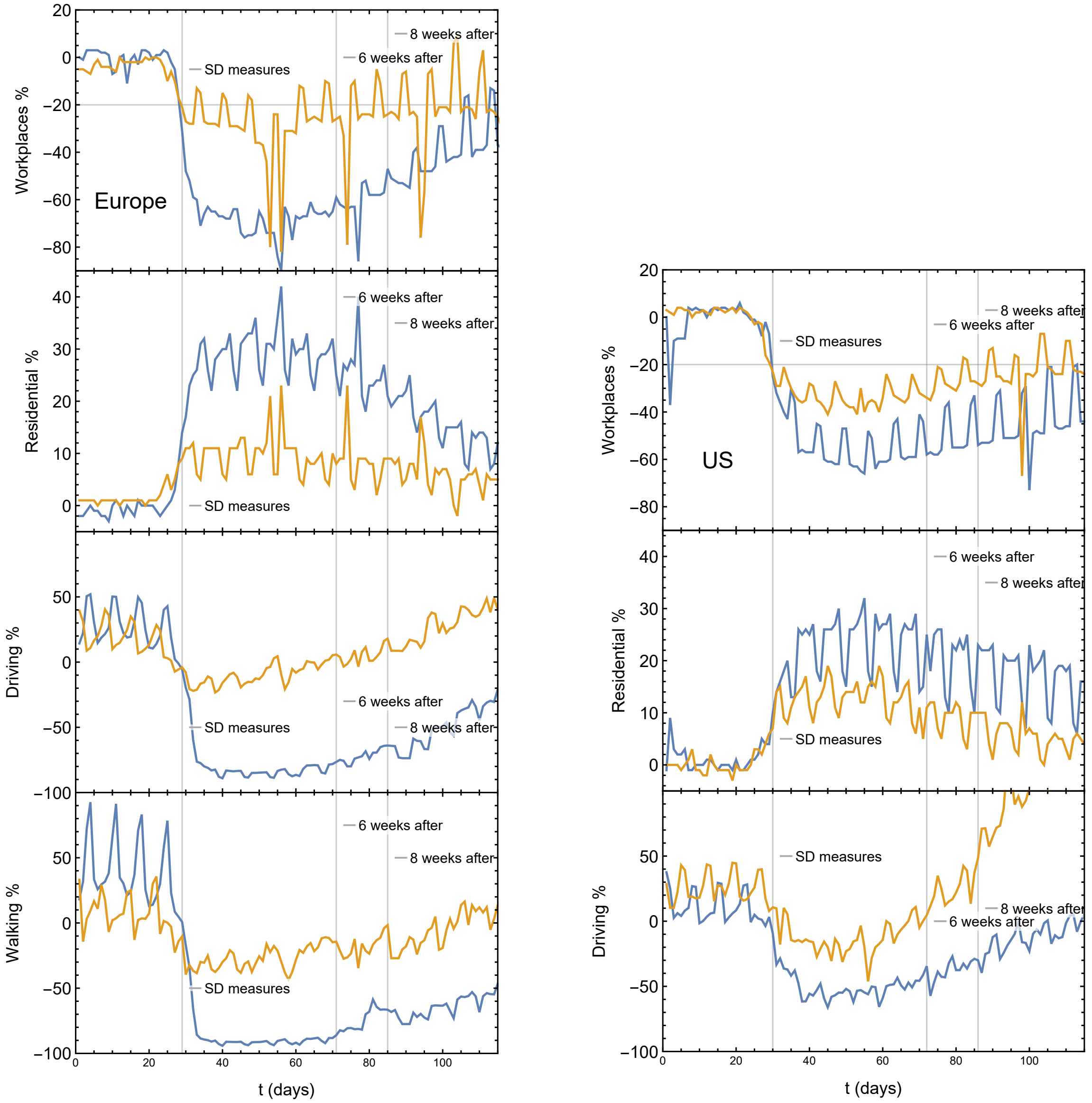

Figure S1. Raw Google and Mobility data. In these plots we show a sample of raw Google and Apple mobility data used in this work, for Europe (left) and the US (right). The time scale is shifted so that the beginning of the social distancing, defined by a 20% drop in Google's Workplace and indicated by the first vertical grey line, coincides for all countries and states. The other two vertical lines mark the end of the 6 and 8 week averaging periods respectively. We show the respective HM region in orange and the LM one in blue: for Europe, Sweden (orange) and Spain (blue); for the US, Wyoming (orange) and New York (blue).

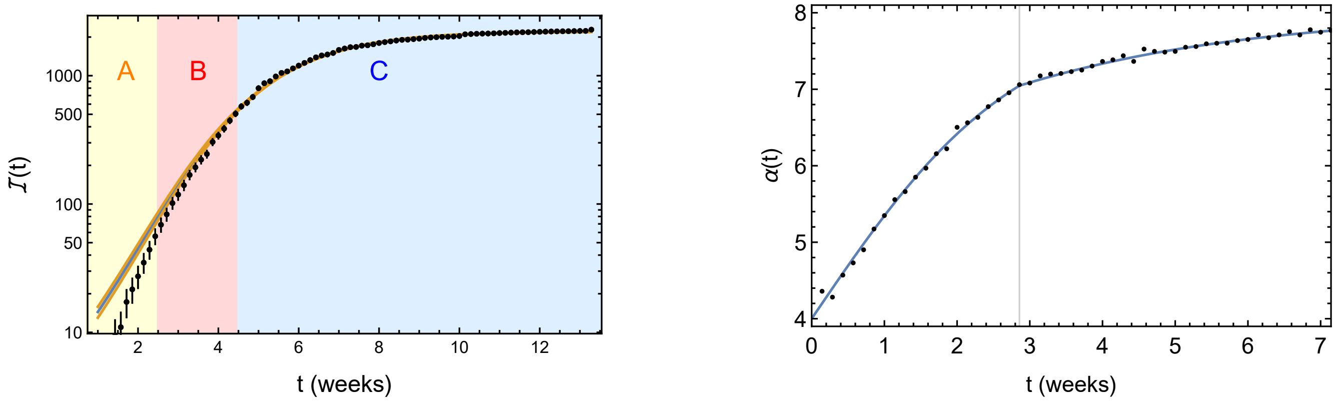

Figure S2. Temporal anatomy of the first wave epidemic data. Left panel: schema of the 3 temporal regions defined in the text. A refers to the pre-measure time, B occurs between the start of the social distancing and the change in γ, C covers the later times, after the measure effects occur. The duration of B is defined as Δt. Right panel: generating function (solid) and sample of the simulated points for the two-gamma model.

Fit parameters: two-gamma function parameters in Europe						
Country	ISO	a	γ_B	γ_C	b	Δt
France	FRA	7.663(9)	0.715(13)	0.64(2)	$1.65(4) \times 10^3$	2.6(2)
Italy	ITA	8.304(9)	0.591(6)	0.386(8)	215(3)	2.88(4)
Spain	ESP	8.483(9)	0.79(1)	0.62(2)	$2.68(5) \times 10^3$	2.74(8)
United Kingdom	GBR	8.339(4)	0.549(9)	0.363(3)	429(9)	2.78(5)
Germany	DEU	7.637(15)	0.726(13)	0.57(3)	$1.75(4) \times 10^3$	2.84(11)
Denmark	DNK	8.06(11)	0.38(2)	0.18(2)	24(1)	4.60(6)
Switzerland	CHE	8.158(7)	0.80(1)	0.71(3)	$2.53(4) \times 10^3$	2.8(2)
Sweden	SWE	8.51(11)	0.353(12)	0.26(2)	39(1)	3.57(8)
Slovakia	SVK	6.04(7)	0.56(25)	0.43(3)	$5(2) \times 10^2$	1.4(1.2)
Portugal	PRT	7.95(1)	0.80(2)	0.548(14)	$6.7(4) \times 10^3$	2.61(8)
Poland	POL	6.25(3)	0.622(12)	0.416(16)	$1.70(5) \times 10^3$	3.07(5)
Ireland	IRL	8.70(4)	0.52(2)	0.26(4)	337(15)	6.26(9)
Netherlands	NLD	7.900(5)	0.67(3)	0.528(7)	$1.18(5) \times 10^3$	1.91(14)
Romania	ROU	7.04(2)	0.557(13)	0.34(1)	$7.0(3) \times 10^2$	3.87(7)
Bulgaria	BGR	6.15(2)	0.86(31)	0.32(8)	$1.9(7) \times 10^4$	1.3(2)
Croatia	HRV	6.296(8)	1.04(4)	0.672(15)	$1.07(7) \times 10^5$	1.89(7)
Norway	NOR	7.42(4)	0.62(2)	0.32(4)	349(6)	3.54(5)
Austria	AUT	7.462(12)	1.060(17)	0.75(5)	$6.1(2) \times 10^4$	2.35(7)
Hungary	HUN	5.972(11)	0.95(22)	0.54(1)	$8(3) \times 10^4$	1.5(2)
Serbia	SRB	7.44(3)	0.641(14)	0.55(5)	$2.18(8) \times 10^3$	5.14(18)
Belgium	BEL	8.481(17)	0.585(11)	0.62(4)	335.15(8)	3.7(6)

Table T2. First wave fits for the two-gamma model for Europe. Outcome of the two-gamma fits for the 5 parameters a, γ_B, γ_C, b and Δt. The errors refer to a 95% CL.

Fit parameters: first wave and 6-week average mobility variations in the US							
State		a	γ	b	Work.	Res.	Driv.
Alabama	AL	7.92(5)	0.40(2)	74(4)	−35	14	−23
Alaska	AK	6.196(7)	0.89(2)	$2.1(2)\times10^4$	−36	14	−23
Arizona	AZ	8.28(7)	0.266(12)	17.5(6)	−40	15	−36
Arkansas	AR	7.72(9)	0.37(2)	63(3)	−32	11	−20
California	CA	8.44(4)	0.243(8)	12.3(5)	−46	20	−48
Colorado	CO	8.01(1)	0.331(6)	31(1)	−47	18	−46
Connecticut	CT	9.124(4)	0.413(6)	73(3)	−45	19	−40
Delaware	DE	7.577(7)	0.399(5)	121(4)	−43	17	−35
Florida	FL	9.76(2)	0.335(14)	20(2)	−42	17	−46
Georgia	GA	11.21(2)	0.316(9)	16.3(7)	−41	16	−32
Hawaii	HI	6.578(2)	0.919(11)	$2.81(8)\times10^4$	−45	20	−62
Idaho	ID	7.751(16)	0.71(4)	$2.2(3)\times10^3$	−38	12	−15
Illinois	IL	8.93(3)	0.322(8)	34(2)	−45	19	−38
Indiana	IN	8.312(12)	0.331(6)	35(2)	−41	16	−30
Iowa	IA	9.721(13)	0.415(7)	169(8)	−34	14	−22
Kansas	KS	9.12(7)	0.335(17)	53(4)	−37	14	−29
Kentucky	KY	6.71(3)	0.395(13)	120(6)	−39	14	−26
Louisiana	LA	8.424(11)	0.82(3)	$8(1)\times10^3$	−39	15	−46
Maine	ME	7.237(11)	0.195(3)	6.7(2)	−39	15	−35
Maryland	MD	10.170(7)	0.293(3)	21.2(5)	−47	20	−40
Massachusetts	MA	10.110(3)	0.405(4)	64(2)	−51	22	−48
Michigan	MI	10.820(9)	0.359(8)	14.6(6)	−51	20	−46
Minnesota	MN	8.727(11)	0.376(6)	137(6)	−43	19	−41
Mississippi	MS	7.88(2)	0.324(7)	36(1)	−34	13	−20
Missouri	MO	7.11(2)	0.447(18)	132(8)	−38	14	−32
Montana	MT	4.390(5)	1.18(3)	$1.1(7)\times10^5$	−36	13	−15
Nebraska	NE	8.75(8)	0.409(7)	233(12)	−33	14	−20
Nevada	NV	7.08(2)	0.467(16)	149(9)	−50	18	−38
New Hampshire	NH	8.704(4)	0.285(2)	18.8(3)	−42	18	−37
New Jersey	NJ	11.340(5)	0.493(7)	127(4)	−52	23	−47
New Mexico	NM	8.06(2)	0.346(7)	59(2)	−39	15	−33
New York	NY	12.530(3)	0.514(6)	110(4)	−53	22	−51
North Carolina	NC	10.73(3)	0.1100(16)	3.55(3)	−38	14	−28
North Dakota	ND	5.22(1)	0.358(6)	151(6)	−32	16	−25
Ohio	OH	9.993(14)	0.310(7)	21.4(7)	−42	16	−30
Oklahoma	OK	6.45(3)	0.345(14)	45(3)	−36	13	−27
Oregon	OR	6.68(2)	0.53(2)	$2.8(3)\times10^2$	−42	15	−37
Pennsylvania	PA	9.8(1)	0.387(9)	45(3)	−46	19	−43
Rhode Island	RI	7.155(5)	0.464(6)	$2.7(1)\times10^2$	−43	18	−38
South Carolina	SC	9.21(3)	0.376(15)	39(3)	−36	13	−25
South Dakota	SD	6.041(12)	0.93(2)	$2.1(2)\times10^5$	−31	14	−13
Tennessee	TN	6.99(3)	0.290(9)	25(1)	−38	14	−30
Texas	TX	9.79(4)	0.379(16)	48(3)	−41	17	−38
Utah	UT	9.69(4)	0.320(12)	20.4(7)	−41	16	−23
Vermont	VT	4.662(5)	1.03(2)	$1.6(2)\times10^5$	−47	18	−48
Virginia	VA	10.64(1)	0.264(3)	16.0(4)	−42	17	−35
Washington	WA	7.896(13)	0.382(9)	18(2)	−48	18	−32
West Virginia	WV	8.04(4)	0.33(2)	26(2)	−37	13	−28
Wisconsin	WI	8.93(4)	0.204(6)	7.7(2)	−39	17	−31
Wyoming	WY	7.60(4)	0.296(15)	20(1)	−33	13	−10

Table T3. First wave fits and average mobility reductions in the US. Same as Table T1.

Fit parameters: two-gamma function parameters in the US						
State		a	γ_B	γ_C	b	Δt
Alaska	AK	6.219(6)	1.28(8)	0.80(2)	$2.9(3) \times 10^6$	1.52(8)
Arizona	AZ	8.87(7)	0.48(3)	0.186(6)	$3.7(3) \times 10^3$	2.87(6)
Arkansas	AR	7.9(2)	0.9(4)	0.33(3)	$2.2(8) \times 10^4$	1.2(3)
California	CA	8.80(5)	0.41(3)	0.179(6)	$1.5(2) \times 10^2$	3.6(1)
Colorado	CO	8.056(9)	0.63(6)	0.301(5)	$1.7(2) \times 10^3$	2.7(2)
Connecticut	CT	9.156(4)	0.480(8)	0.333(7)	215(8)	5.3(2)
Delaware	DE	7.596(7)	0.59(5)	0.379(6)	$2.1(3) \times 10^3$	3.6(2)
Florida	FL	10.16(5)	0.51(3)	0.172(9)	$2.6(2) \times 10^2$	3.55(7)
Georgia	GA	11.40(2)	0.45(2)	0.228(6)	126(6)	3.86(8)
Hawaii	HI	6.586(7)	0.91(2)	0.7(2)	$2.89(9) \times 10^4$	4.9(4)
Idaho	ID	7.95(2)	1.23(5)	0.31(2)	$2.6(3) \times 10^6$	2.66(3)
Illinois	IL	9.07(3)	0.53(4)	0.283(6)	$6.3(6) \times 10^2$	2.8(1)
Indiana	IN	8.378(8)	0.62(3)	0.293(4)	$1.9(3) \times 10^3$	3.01(6)
Iowa	IA	10.3(1)	0.33(2)	0.18(2)	62(2)	7.38(5)
Kansas	KS	9.14(9)	0.6(6)	0.33(2)	$1(1) \times 10^3$	1.9(1.3)
Kentucky	KY	6.66(9)	0.40(3)	0.5(1)	130(6)	6.9(6)
Louisiana	LA	8.65(3)	0.91(3)	0.31(3)	$3.7(2) \times 10^4$	3.35(4)
Maine	ME	7.279(9)	0.53(6)	0.180(3)	$5.7(7) \times 10^3$	2.6(2)
Maryland	MD	10.197(6)	0.48(3)	0.277(3)	$3.3(3) \times 10^2$	3.7(2)
Massachusetts	MA	10.138(4)	0.441(6)	0.339(6)	120(3)	5.8(2)
Michigan	MI	10.902(6)	0.47(2)	0.274(4)	66(2)	2.55(7)
Minnesota	MN	8.695(8)	0.23(2)	0.400(5)	16(1)	4.9(2)
Mississippi	MS	8.00(2)	0.50(4)	0.284(6)	$4.0(3) \times 10^2$	2.9(2)
Missouri	MO	7.32(3)	0.66(3)	0.30(2)	$2.9(2) \times 10^3$	3.07(7)
Montana	MT	4.389(6)	1.18(3)	0.767(0)	$1.33(9) \times 10^6$	7.7(0)
Nebraska	NE	8.748(9)	0.36(9)	0.411(8)	$1.2(4) \times 10^2$	4(2)
Nevada	NV	7.24(2)	0.73(3)	0.333(9)	$5.2(4) \times 10^3$	2.89(5)
New Hampshire	NH	8.705(4)	0.284(3)	0.095(0)	19.5(3)	19(0)
New Jersey	NJ	11.361(3)	0.71(2)	0.447(4)	$2.3(1) \times 10^3$	2.83(6)
New Mexico	NM	8.18(3)	0.49(3)	0.306(6)	$4.8(4) \times 10^2$	3.6(1)
New York	NY	12.529(4)	0.513(7)	0.0855(0)	115(3)	18(0)
North Carolina	NC	10.88(3)	0.28(3)	0.103(1)	43(4)	3.9(2)
North Dakota	ND	5.26(2)	0.45(3)	0.331(8)	$7.0(8) \times 10^3$	4.9(3)
Ohio	OH	10.18(3)	0.351(7)	0.222(8)	46(2)	5.29(9)
Oklahoma	OK	6.71(3)	0.68(3)	0.230(7)	$4.7(4) \times 10^3$	3.17(5)
Oregon	OR	7.02(5)	0.70(3)	0.30(2)	$2.0(2) \times 10^3$	2.80(6)
Pennsylvania	PA	9.942(5)	0.60(2)	0.296(4)	$9.5(4) \times 10^2$	3.88(4)
Rhode Island	RI	7.195(4)	0.533(7)	0.384(6)	$8.4(3) \times 10^2$	5.47(8)
South Carolina	SC	9.57(5)	0.50(2)	0.23(2)	$2.7(2) \times 10^2$	3.17(7)
South Dakota	SD	6.04(2)	0.93(3)	0.333(0)	$2.4(2) \times 10^5$	8.7(0)
Tennessee	TN	7.3(2)	0.25(2)	0.18(4)	18.0(7)	6.6(3)
Texas	TX	10.56(9)	0.43(2)	0.19(1)	147(5)	3.79(4)
Utah	UT	10.01(4)	0.48(6)	0.232(6)	208(8)	2.89(5)
Vermont	VT	4.75(2)	0.98(3)	0.36(6)	$1.06(5) \times 10^6$	3.72(6)
Virginia	VA	10.67(1)	0.43(6)	0.256(4)	$1.7(3) \times 10^2$	3.4(3)
Washington	WA	8.20(2)	0.349(5)	0.204(5)	15.3(2)	2.36(4)
West Virginia	WV	9.4(5)	0.35(4)	0.09(2)	67(5)	4.15(8)
Wisconsin	WI	9.13(3)	0.45(4)	0.176(4)	$2.2(2) \times 10^2$	2.6(1)
Wyoming	WY	7.9(3)	0.25(4)	0.17(7)	13.0(7)	7.3(5)

Table T4. First wave fits for the two-gamma model for the US. Same as Table T2.
